# Outcomes of pregnant ICU patients with severe COVID-19 pneumonia in Qatar during the three waves of the COVID-19 pandemic: A retrospective cohort study

**DOI:** 10.5339/qmj.2025.12

**Published:** 2025-02-27

**Authors:** Layla J.M. Kily, Sohel M.G. Ahmed, Tamam A.K.M. Alhusban, Mohammed J. Orompurath, Marcus D. Lance, Mogahed I.H. Hussein, Salwa M. Abuyaqoub, Huda A. Saleh, Eynas Abdalla, Santhosh Gopalakrishnan, Hilal Al-Rifai, Mohamed Hilani, Hayat Elfil

**Affiliations:** ^1^Department of Anesthesiology, ICU and Perioperative Medicine, Hamad General Hospital, Doha, Qatar; ^2^Qatar University College of Medicine, Doha, Qatar; ^3^Department of Anesthesiology, ICU and Perioperative Medicine, Women's Wellness and Research Centre, Doha, Qatar; ^4^Clinical Trial Unit, Academic Health System, Hamad Medical Corporation, Doha, Qatar; ^5^Department of Anesthesiology, ICU and Perioperative Medicine, Aga Khan University Medical College, Nairobi, Kenya; ^6^Department of Obstetrics and Gynaecology, Women's Wellness and Research Centre, Doha, Qatar; ^7^Neonatal-Perinatal Medicine Department, Women's Wellness and Research Centre, Doha, Qatar *Correspondence: Zeynel Abidin Sayiner. Email: Ikily@hamad.qa

**Keywords:** COVID-19, intensive care unit, vaccination, fetal outcome, non-invasive ventilation, high flow nasal cannula

## Abstract

**Introduction:**

Pregnant women are considered a high-risk group for COVID-19 infection/pneumonia as they are known to be more vulnerable to viral infections. They require close monitoring and appropriate timely intervention to minimize the impact on both the mother and the fetus. Although the more prevalent Omicron variant led to fewer severe infections and fewer intensive care unit (ICU) admissions globally during the third wave, the effect on pregnant women and pregnancy outcomes was unknown. The vaccination campaign was thoroughly established by the third wave of the pandemic in Qatar. This retrospective descriptive cohort study investigates the characteristics, hospital stay, interventions, vaccination status, and fetal and maternal outcomes of patients admitted to the ICU with severe COVID-19 pneumonia during each of the three COVID-19 waves in Qatar.

**Methods:**

The inclusion criteria were all pregnant patients with a positive polymerase chain reaction antigen test result and/or defined radiological changes at the time of admission that subsequently required admission to the ICU for 24 hours or more. Data were collected from the medical records and chart reviews of patients admitted to Hamad Medical Corporation with COVID-19 pneumonia from March 1, 2020 to February 28, 2022.

**Results:**

The study included a total of 54 pregnant women. In contrast, during the third wave, the number of patients admitted to the ICU was significantly less than in the first wave. The mean gestational age at presentation for each of the three waves was 213.5, 212, and 245 days, respectively. No pregnant women were vaccinated during the first two waves. However, during the third wave, 90.9% of patients admitted to the ICU were vaccinated. The average length of stay in hospital was (mean ± standard deviation) 22.0 ± 27.6, 15.5 ± 7.8, and 5.0 ± 6.3 days for each of the waves, respectively, and the average length of ICU stay was 13.4 ± 20.9, 6.3 ± 5.5, and 3 ± 2.5 days, respectively. The most common chest X-ray finding on admission was bilateral infiltrates. During the third wave, only one patient required a high-flow nasal cannula. As the severity of the disease increased, the patients received more invasive respiratory support and had a higher likelihood of a preterm delivery. Vaccination status correlated with a significantly higher birth weight (mean weight 3.14 kg). However, it was not associated with better maternal outcome.

**Conclusion:**

This extension study of the COVID-19 patients admitted to the ICU in Qatar during all three waves suggests that those admitted to the ICU with COVID-19 pneumonia are more likely to require close monitoring and appropriate interventions to minimize adverse outcomes for both the mother and the fetus. Our data may suggest that vaccination in these patients may contribute to reducing the use of respiratory support modalities for those admitted to the ICU and shortening the length of hospital stay. Overall, there was no statistical significance between vaccination and maternal outcome.

## Introduction

As of February 2024, the highly infectious SARS-CoV-2 RNA virus has reportedly infected almost 775 million people globally,^1^ of which 514,500 cases have been reported in Qatar so far.^1^ People with the SARS-CoV-2 virus infection may either be asymptomatic or have a variety of symptoms that show a variable course of the COVID-19 disease.^2–5^

Pregnant patients are at a higher risk of increased disease severity, including intensive care unit (ICU) admission and invasive ventilation, as well as the need for extracorporeal membrane oxygenation (ECMO) compared to age-matched non-pregnant patients. Furthermore, recent data from the Centers for Disease Control and Prevention (CDC) has shown an increased risk of COVID-19-related mortality compared to non-pregnant women of the same age.^6^ In addition, they are more likely to be symptomatic,^7^ with some studies suggesting that they were more likely to have severe illness and higher mortality^8^ than non-pregnant women. Morbidity is another concern as COVID-19 positive mothers are more likely to have preeclampsia, eclampsia, hemolysis, elevated liver enzymes, and low platelets (HELLP) syndrome, secondary infections, as well as higher rates of neonatal intensive care unit (NICU) admission, prematurity, and neonatal morbidity.^9,10^

There is growing evidence of additional risk factors among pregnant women with COVID-19 that are associated with higher morbidity, such as older age, obesity, pre-existing diabetes, gestational diabetes, and hypertension.^11,12^ However, in Qatar, a recent study found no significant association between pregnancy outcomes and the need for respiratory support,^11^ although pregnant women had more respiratory symptoms such as shortness of breath compared to non-pregnant women.^7^ This finding may be attributed to the fact that Qatar is a higher-income country and has better healthcare services, as higher-income countries are reported to have fewer adverse outcomes.^13,14^

Since the COVID-19 vaccine became available, research has been conducted to compare maternal and neonatal outcomes in relation to vaccination status, previous COVID-19 infection, and current infectious status. These data largely relate to the second (predominantly Delta variant) and third (predominantly Omicron variant) waves of infection when the COVID-19 vaccine became available, as well as differences in the pathogenicity and infectivity of the different strains, leading to various conflicting results. One study found that vaccinated pregnant women who contracted COVID-19 were similarly likely to have small-for-gestational-age fetuses compared with unvaccinated women.^15^ In contrast, most pregnant women with COVID-19 had a mild disease course with good outcomes.^16^ However, there are conflicting results for women vaccinated against COVID-19. Some studies show no significant differences in maternal and fetal outcomes between vaccinated and unvaccinated women.^15,17^ Another study found that the rate of SARS-CoV-2 infections was significantly lower in vaccinated pregnant women than in unvaccinated pregnant women.^18^ In Qatar, wave 1 occurred between March 1, 2020 and March 31, 2021, wave 2 between April 1, 2021 and June 30, 2021, and wave 3 between December 1, 2021 and February 28, 2022. During the pandemic, the virus continued to mutate until the third wave and transmissibility increased due to the high number of mutations, particularly in the spike protein.^19^ The Omicron-dominant variant was more infectious, but was considered less pathogenic with a milder course of the disease.^19^ Previous infection and vaccination did not provide protection against infection with the Omicron variant. However, it was observed to reduce disease severity and the need for hospitalization.^19,20^

In general, pregnant women were less likely to take the vaccine early after its introduction due to vaccine hesitancy and long-term safety concerns.^17,21^ The effects of this variant on the population, including non-pregnant women, were observed to vary.^22–24^ The aim of this retrospective descriptive cohort study was to describe the characteristics, respiratory support modalities, and both fetal and maternal outcomes of pregnant patients admitted to the ICU with severe COVID-19 infection during the three waves of the pandemic in Qatar in the context of vaccination status, and may support future guidelines and strategies for managing and predicting outcomes in severe disease.

## Methodology

As an extension to our previous study,^11^ this retrospective descriptive cohort study with Institutional Review Board (IRB) number MRC-01-23-770 examines the three waves during the period from March 1, 2020 to February 28, 2022. We compare characteristics, hospital stay, interventions, and neonatal and maternal outcomes of pregnant patients with COVID-19 admitted to the ICU in Qatar with special regard to the vaccination status.

Data were collected from the charts of our electronic health system (Cerner-Oracle) and entered into Excel (Microsoft Excel) spreadsheets and analyzed using IBM SPSS 22 (IBM, Chicago, IL, USA). The IRB of the Medical Research Centre (MRC), Hamad Medical Corporation (Protocol No. MRC-01-21-790) waived the need for ethical approval due to the nature of the study.

The main outcomes of the study were to find a correlation between various characteristics of the pregnant population with severe COVID-19 infection, including the respiratory support modalities used and the outcomes of both the mothers and the babies born thereafter. Other factors examined were vaccination status and its association with the respiratory support modality used and the patient's subsequent hospital course. Neonatal outcomes were characterized by gestational age and weight at birth, APGAR (Appearance, Pulse, Grimace, Activity, and Respiration) scores at 1 and 5 min, and whether the neonate was admitted to the NICU.

The study data were collected as continuous and nominal data. Normality was analyzed using the Shapiro–Wilk normality test. Descriptive statistics were performed on continuous data, with central tendency represented as mean (standard deviation) or median (interquartile range) based on normal distribution. Nominal data with or without multiple responses are represented in frequency or count with valid percentages.

For normally distributed variables, one-way ANOVA was used to test the mean difference between different COVID-19 wave cohorts and the alternative Kruskal–Wallis test was used for non-parametric data variables. The risk estimate was calculated as a relative risk or odds ratio. Logistic regression was performed to determine the combined effects of respiratory distress, number of respiratory modalities used, and severity score on the likelihood of patients having a full-term delivery. A *p* value  < 0.05 was considered statistically significant.

Inclusion criteria were all pregnant women hospitalized with COVID-19 with either a positive polymerase chain reaction (PCR) antigen test result and/or defined radiological changes at the time of admission and who subsequently required admission to the ICU.

Exclusion criteria were pregnant patients who were not admitted to hospital or the ICU or were discharged home within 24 hours despite a positive PCR antigen test. Patients whose respiratory data were missing or incomplete were included for characteristic analysis when available, but were later removed and excluded from the ad hoc analysis.

## Results

A total of 54 pregnant women admitted to the ICU were included in the study during each of the three waves (*n* = 32, *n* = 11, and *n* = 11, respectively). Demographic data and characteristics of their presentation were collected, as well as vaccination status, presence of any comorbidities, maternal blood group, blood parameters at ICU admission, mode of delivery and anesthesia (if applicable) at the time of delivery, and neonatal parameters including gestational age at presentation, birth weight, gestational age at birth, APGAR scores, and NICU admission during each of the three waves (Table 1).

Most women (*n* = 32) were admitted to the ICU with severe COVID-19 pneumonia during the first wave, with less number of patients during the second and third waves (*n* = 11 and *n* = 11, respectively). The mean gestational age at presentation for each of the three waves was 213.5, 212, and 245 days, respectively. Most women were at least gravida 2 and had a mean age of 32.62 (21–43) years.

Qatar is home to a diverse multinational population, which makes up the majority of the population.^25^ To simplify the analysis of which nationalities presented with severe COVID-19 pneumonia, as Qatar and the surrounding Middle East/Gulf region consisting mainly of Arabic-speaking natives, the nationalities were nominally divided into Arabic-speaking and non-Arabic-speaking nationalities. Therefore, it was found that there was no statistically significant difference between the nationalities that developed severe COVID-19 pneumonia, with more Arabic-speaking nationals affected during the first and third waves (Appendix 1).

**Figure 1.**
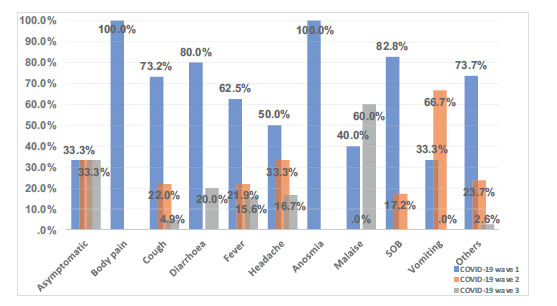
Frequency percentage of symptoms at presentation among patients admitted to the ICU during each of the three waves (*n* = 49). SOB: shortness of breath.

The length of hospital stay was significantly shorter during the third wave, which correlates with the severity of illness and respiratory distress, indirectly measured by the number and invasiveness of the respiratory support modality needed for the patient (Table 2 and Figure 1). During the third wave, 91% of the patients admitted to the ICU were vaccinated, while patients in the first and second waves were unvaccinated. The chi-square test showed no association between vaccination and pregnancy outcome. “Vaccination” in our study was defined as having completed two doses of one of the two vaccines offered and available in Qatar during the study period (Pfizer-BioNTech Comirnaty® or Moderna vaccine SPIKEVAX®). Although a third booster dose was initially introduced for patients above the age of 65 or immunocompromised, as well as those with certain medical conditions, it was not made available to the general population, including pregnant women, until 14 days before the start of the third wave. At the time, Qatar had defined “fully vaccinated” as having received two approved vaccine doses, with the second dose received within the last nine months. Subsequently, no pregnant woman took a third booster dose during the study. Patients who completed their vaccination course of two doses and presented within 6 months of the latest dose were analyzed for timing of vaccine doses using the Kruskal–Wallis test, and there was no statistical significance between vaccination time and severity of respiratory distress.

Interestingly, there were significant chest X-ray (CXR) findings of bilateral infiltrates at presentation (Table 2), which were consistent across all three waves. This is contrary to expectations as shortness of breath was the predominant symptom during the first wave. Despite these initial CXR findings, no patient presented with shortness of breath during the third wave.

During the first wave, a greater variety of symptoms were described by patients, which decreased with each progressive wave. To simply the analysis, only the first five main symptoms complained of by the patient were considered, while other symptoms such as nausea, runny nose, sore throat, and abdominal pain were documented as “others” (Figure 1).

Fever was common throughout, and although cough was frequent during the first and second waves, only a few patients complained of it as a presenting symptom during the third wave. Anosmia was not a feature in any of the patients requiring ICU support, with only one documented patient complaining of this symptom during the first wave. Malaise was a predominant symptom during the third wave. Asymptomatic patients were found to be COVID-19 positive on elective admission screening and required admission to the ICU during their hospital stay (Figure 1).

The O positive blood group was the most commonly affected blood group, but was not statistically significant in all three waves (Table 2). Across all waves, anemia was a common finding at presentation with a mean hemoglobin (Hb) level of 11.2, 11.1, and 10.8 g/dL, respectively. The average CRP (C-Reactive Protein) at the time of presentation decreased with each subsequent wave. Viral load, as measured by the cycle threshold (CT) value, ranged from 19.0 to 22.6.

More women had pre-existing comorbidities during the first wave than in the second and third waves (Appendix 1). During the first wave, 43.8% of women had diabetes (type 1, 2, or gestational), while no women had diabetes in the second and third waves. Women in the first wave had comorbidities such as hypothyroidism and more were obese and asthmatic compared to the other waves.

For fetal outcomes, there was statistical significance in birth weight, with babies born during the third wave having a higher birth weight. However, there was no statistically significant difference in gestational age at birth across the three waves.

There was no difference in APGAR scores at 5 min and no difference between NICU admission rates during the three waves. There was no statistical difference between modes of delivery during the three waves, although more women received general anesthesia for lower segment cesarean section (LSCS) delivery during the first wave (Appendix 1). The chi-square analysis revealed no statistical significance between vaccination status and maternal outcomes.

**Figure 2.**
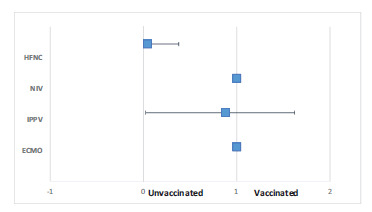
Graph showing the relative risk of respiratory support according to vaccination status (*n* = 53). There is overlap in the confidence interval for IPPV applied in vaccinated and unvaccinated patients, indicating no statistical significance between vaccinated and unvaccinated patients. HFNC: high-flow nasal cannula, NIV: non-invasive ventilation, IPPV: intermittent positive pressure ventilation, ECMO: extracorporeal membrane oxygenation.

Further regression analysis was performed to analyze the relationship and relative risk of vaccination status versus respiratory support modality used across the three waves (Table 3 and Figure 2). One patient was excluded from this analysis because she was admitted to the ICU due to diabetic ketoacidosis rather than severe COVID-19 pneumonia and therefore did not require any of the advanced respiratory support modalities such as high-flow nasal cannula (HFNC), non-invasive ventilation (NIV), or intermittent positive pressure ventilation (IPPV), using only a maximum of 3 L/min via a nasal cannula for a limited time.

The relative risk of requiring a particular type of respiratory support modality according to vaccination status during all three waves showed a statistical difference in the use of HFNC during the third wave. The odds ratio for HFNC use was 0.043, indicating statistical significance that vaccination status reduced the odds of HFNC use by 96%. The odds of requiring IPPV decreased by almost 82% if the patient was vaccinated (odds ratio 0.188), although this was not statistically significant at a 95% confidence interval.

In unvaccinated patients, NIV was used in the form of either CPAP (Continuous Positive Airway Pressure) ventilation or BiPAP (Bilevel Positive Airway Pressure) ventilation, or both, with an odds ratio of 0.53 representing 47% of vaccinated patients requiring NIV.

The logistic regression model was statistically significant (*χ* ^2^(3) = 7.53, *p* = 0.05). The model accounted for 19.0% (Nagelkerke R^2^) of the variance in delivery outcome and was correctly classified in 74.0% of cases (Table 4). It was found that each additional modality used was associated with a reduction in the likelihood of patients having a full-term birth. Specifically, with each additional unit in respiratory support modality, the likelihood of patients having a full-term delivery decreased by 31% (increasing the likelihood of preterm birth by 69%), assuming all other predictors remained constant.

## Discussion

In the present study, we compared the clinical courses of pregnant patients admitted with severe COVID-19 pneumonia according to their vaccination status. The COVID-19 vaccine was first approved for emergency use in Qatar in December 2020^26^ and had not yet been taken by any of our patients until the second wave. Vaccination was defined as having received both doses of one of the only two nationally approved vaccines at the time (Pfizer-BioNTech Comirnaty® or Moderna SPIKEVAX® vaccine).

By the third wave, 91% of our patients admitted to the ICU with severe COVID-19 infection had been vaccinated. However, they were seven times more likely to have HFNC support and three times more likely to require IPPV if they were unvaccinated (Table 3). This suggests that vaccination may provide some protection against the need for additional respiratory support modalities.

Regardless of the vaccination status, the length of hospital stay was significantly shorter in the third wave. Moreover, it was observed that there may be improved fetal outcomes due to fewer preterm births and higher birth weight. This finding varies across countries, as one recent study in the UK found that pregnant women, despite vaccination, were three times more likely to have a preterm birth with severe Omicron infection than unvaccinated mild infection with the wild-type variant.^27^ However, another study found a reduction in the rate of preterm births and stillbirths in vaccinated mothers^28^ and another found no difference in birth weight between vaccinated and unvaccinated mothers.^29^

The Omicron variant was the dominant variant during the third wave, which is more likely to cause reinfection regardless of previous natural immunity, vaccine-induced immunity, or a hybrid of both due to immune escape.^19,30–32^ Although our study did not measure for evidence of previous infection before vaccination, hybrid immunity provides more protection against infection than natural immunity or vaccination alone.^33^ Effective immunity established after the second vaccination dose was expected to provide protection against hospitalization and death for six months after the second dose.^34^ We did not measure whether vaccination occurred during pregnancy or the time interval between vaccination doses. However, we found that 45.5% of vaccinated patients presented within six months of the second dose. This corresponded to the number of patients admitted to the ICU whose second dose was more than six months ago.

In our cohort, pregnant women showed a wide variation in their main symptoms during each of the three waves. This result is comparable to international findings. For example, in one study in Wuhan, China, after the initial outbreak, pregnant women were found to have different symptoms than non-pregnant women.^35^ A study in Serbia comparing clinical manifestations and clinical outcomes during their four waves of the pandemic found that certain groups of symptoms were more strongly associated with each virus variant. For example, the Omicron variant caused more sore throats, coughs, headaches, and less severe clinical presentations.^36^ Furthermore, symptoms were associated with comorbidities. For example, pregnant patients with a higher BMI were more likely to be symptomatic for COVID-19^37^ and also more at risk factors for developing a severe disease.^11,38^

Several studies have shown that maternal comorbidities influence maternal and neonatal outcomes, including a higher risk of LSCS and stillbirth.^39^ During the first wave, there were a significant number of women who had diabetes, and this finding is consistent with other studies that found women with gestational diabetes were at increased risk of severe COVID-19 infection as well as adverse neonatal outcomes.^40^ Overall, there were fewer pre-existing comorbidities during the second and third waves (Appendix 1).

In our study, fewer patients were admitted to the ICU during the third wave, which was dominated by the Omicron variant, compared to the first wave. Although this variant was more infectious, it was associated with milder disease and fewer hospitalizations compared to global findings.^31,36^ Additionally, during the third wave, no patients required RRT (Renal Replacement Therapy) or ECMO and there was no fetal or maternal mortality.

We propose that although there appears to be no difference in maternal outcomes, there may be an association between pregnancy outcome and vaccination status. During each wave, differences were observed between the length of hospital stay and the number of patients admitted with severe disease and requiring ICU support. This can also be attributed to viral pathogenicity, as in general more patients were infected with Omicron, but fewer patients required hospitalization and ICU admission.^31^

## Strengths

To date this is the only study of its kind conducted in Qatar. The relatively small population size did not favor an in-depth analysis of the characteristic variables, which provide a good basis for further future comparative studies.

Data collection from a single center limits survey bias and the variable population affected may allow for generalizability to other regions.

## Limitations

As only a single center was studied, this may limit the diversity of the population and generalizability of the findings and the sample size may also potentially bias the results. There were also a limited number of unvaccinated patients in the third wave that could have provided valuable comparative data.

Confounding variables such as demographic factors or comorbidities that may have influenced the study outcomes were not matched with controls. The retrospective nature of the study and the reliance on pre-existing data may introduce bias that may limit the study.

Further analysis of the timing of vaccine doses, whether taken before or during pregnancy, as well as whether there was any previous documented COVID-19 infection, would have provided more comparative information on whether the timing of the last vaccine doses or the effect of hybrid immunity affected outcome data.

## Conclusion

The SARS-CoV2 virus has a variable presentation in pregnant women in general and during the different waves. In our study, we found that most pregnant women with severe disease were unvaccinated. However, during the third wave with the Omicron-dominant variant, which was considered milder, most women were vaccinated and there was no statistical significance between vaccination and maternal outcome. However, vaccination was associated with decreased requirement and invasiveness of respiratory support modalities such as HFNC. Patients admitted with severe COVID-19 pneumonia had a significantly shorter length of hospital stay during the third wave compared to previous waves.

Our study also suggests that the fetus may benefit from maternal vaccination as it is statistically more likely to be born at full term and have a higher birth weight. There was no overall statistical difference in the use of respiratory support and fetal outcomes.[Table tbl5]

### Competing interests

The authors have no conflicts of interest to declare.

## Figures and Tables

**Table 1.** Characteristic parameters of our cohort at ICU admission according to the three COVID-19 waves (*n* = 54).

Characteristics	Wave 1 *N* = 32	Wave 2 *N* = 11	Wave 3 *N* = 11	*p*

*Mother-related parameters*Gestational presentation (days)*	213.5 (71.25)	212 (63)	245 (120)	0.498

Gestation at birth (days)*	1264 (254.82)	1282.45 (282.37)	1431.81 (164.44)	0.161

Pregnancy outcome^+^				0.1

Full term	14 (48.3)	8 (72.7)	9 (81.8)	

Preterm	15 (51.7)	3 (27.3)	2 (18.2)	

Gravidity^+^				0.172

G1	1 (3.1)	2 (18.2)	2 (18.2)	

≥ G2	31 (96.9)	9 (81.8)	9 (81.8)	

Parity^+^				0.175

0	2 (6.3)	3 (27.3)	2 (18.2)	

P1	8 (25)	3 (27.3)	4 (36.4)	

P2–P4	21 (65.6)	4 (36.4)	3 (27.3)	

≥ P5	1 (3.1)	1 (9.1)	2 (18.2)	

Age**	33.37 {5.25}	30.36 {2.69}	33 {3.41}	0.182

BMI**	32.69 {5.10}	30.88 {6.33}	31.39 {4.62}	0.561

Nationality^+^				0.43

Arabic speaking	17 (53.1)	4 (36.4)	7 (63.6)	

Non-Arabic speaking	15 (46.9)	7 (63.6)	4 (36.4)	

Total hospital stay (days)*	13.50 {9.5}	12 {12}	3 {3}	0.00

ICU stay (days)	6 {7.8}	5 {8}	2 {4}	0.03

Vaccination status^+^				0.00

Unvaccinated	32 (100)	11 (100)	1 (9.1)	

Vaccinated	0	0	10 (90.9)	

*Neonatal parameters*				

Birth weight**	2.88 {1.37}	2.86 {0.98}	3.14 {1.06}	0.00

APGAR score at 5 min**	10 {1.8}	10 {1.3}	10 {0}	0.542

NICU admission				0.736

No	19 (67.9)	8 (72.7)	8 (72.7)	

Yes	9 (32.1)	3 (27.3)	3 (27.3)	


*N*: number of patients,*mean (standard deviation), **median {interquartile range}, ^+^frequency (% within column). The bold values are statistically significant at the 95% confidence level (*p* < 0.05).

**Table 2.** Clinical characteristic parameters for each wave.

Characteristics	Wave 1 *n* = 32	Wave 2 *n* = 11	Wave 3 *n* = 11	*p*

Chest X-ray^+^				0.020

B/I infiltrates	17 (54.8)	2 (20)	5 (55.6)	

Consolidation	0	1 (10)	1 (11.1)	

Pulmonary edema	0	0	1	

Normal	0	0	1	

Others	14 (45.2)	7 (70)	1 (11.1)	

Blood group^+^				0.666

A	1 (3.1)	0	0	

A-	3 (9.4)	0	0	

A^+^	5 (15.6)	2 (18.2)	3 (27.3)	

AB^+^	6 (18.8)	1 (9.1)	1 (9.1)	

B^+^	8 (25)	1 (9.1)	3 (27.3)	

O-	1 (3.1)	0	0	

O^+^	8 (25)	7 (63.6)	4 (36.4)	

Blood parameter				

Hb*	11.24 (1.17)	11.12 (1.19)	10.78 (1.63)	0.465

WBC**	5.8 {4}	6.8 {4.10}	10 {18.68}	0.009

CRP**	56.5 {50.1}	48.7 {38}	24 {45.7}	0.022

Creatinine**	39.5 {15.3}	39 {5}	21.12 {37]	0.071

CT value**	21.70 {6.84}	19 {5.50}	22.60 {8.13}	0.734


*Mean (standard deviation), **median {interquartile range}, ^+^frequency (% within column). The bold values are statistically significant at the 95% confidence level (*p* < 0.05).

B/l: bilateral, Hb: hemoglobin, WBC: white blood cells, CRP: C-reactive protein, CT value: cycle threshold value.

**Table 3.** Relative risk of respiratory support according to vaccination status during all three waves (*n* = 53).

		Applied			Not applied				

Support	*N*	Vaccinated	Not vaccinated	RR	Vaccinated	Not vaccinated	RR	*p*	Odds ratio

HFNC	53	1	31	7.209 (1.113–46.71)	9	12	0.31 (0.184–0.523)	0.001	0.043 (0.005–0.377)

NIV	51	0	20	1	8	23	0.535 (0.405–0.707)	0.016	1

IPPV	53	1	16	3.721 (0.557–24.865)	9	27	0.698 (0.512–0.950)	0.14	0.188 (0.022–1.620)

ECMO	53	0	2	1	10	41	0.953 (0.893–1.019)	1	1


*N*: number of patients, RR: risk ratio, *p*: probability (statistically significant at 95% confidence level), HFNC: high-flow nasal cannula, NIV: non-invasive ventilation, IPPV: intermittent positive pressure ventilation, ECMO: extracorporeal membrane oxygenation.

**Table 4.** Logistic regression analysis to determine the combined effects of respiratory distress, number of respiratory support modalities used, and severity score on the likelihood of patients having a full-term delivery outcome using the Nagelkerke R^2^ model of the variance.

**Predictor**	**Definition**	***B***	**SE**	**Wald**	**df**	**Sig.**	**Exp(** ***B*** **)**	**95% CI for EXP(** ***B*** **)**	

								**Lower**	**Upper**

Intercept		-0.943	1.558	0.366	1	0.545	0.390		

Respiratory distress	Any modality is applied or not	1.964	1.777	1.221	1	0.269	7.125	0.219	231.896

Number of modalities	Actual number of modalities applied	-1.159	0.470	6.075	1	0.014	0.314	0.125	0.789

Severity score	Based on the number of the modalities applied and length of stay	-0.210	1.468	0.020	1	0.886	0.810	0.046	14.405


A *p* value < 0.05 was considered to be statistically significant.

**Table tbl5:** List of abbreviations

APGAR	Appearance, Pulse, Grimace, Activity, and Respiration

BiPAP	Bilevel Positive Airway Pressure

CDC	Centers for Disease Control and Prevention

COVID-19	Coronavirus Disease-2019

CPAP	Continuous Positive Airway Pressure

CRP	C-Reactive Protein

CT value	Cycle Threshold value

CXR	Chest X-Ray

ECMO	Extracorporeal Membrane Oxygenation

Hb	Hemoglobin

HELLP	Hemolysis, Elevated Liver Enzymes, and Low Platelets

HFNC	High-Flow Nasal Cannula

ICU	Intensive Care Unit

IPPV	Intermittent Positive Pressure Ventilation

LSCS	Lower Segment Cesarean Section

NICU	Neonatal Intensive Care Unit

NIV	Non-invasive Ventilation

PCR	Polymerase Chain Reaction

RNA	Ribonucleic Acid

RRT	Renal Replacement Therapy

SARS-CoV-2	Severe Acute Respiratory Syndrome Coronavirus-2

SOB	Shortness of Breath


**Table tbl6:** 

Characteristics	Wave 1 *n*=32	Wave 2 *n*=11	Wave 3 *n*=11	*p*

*Mother-related parameters* Gestational presentation (days)*	213.5 (71.25)	212 (63)	245 (120)	0.498

Gravidity^+^				0.172

G1	1 (3.1)	2 (18.2)	2 (18.2)	

≥ G2	31 (96.9)	9 (81.8)	9 (81.8)	

Parity^+^				0.175

0	2 (6.3)	3 (27.3)	2 (18.2)	

P1	8 (25)	3 (27.3)	4 (36.4)	

P2 to P4	21 (65.6)	4 (36.4)	3 (27.3)	

≥ P5	1 (3.1)	1 (9.1)	2 (18.2)	

Age**	33.37 {5.25}	30.36 {2.69}	33 {3.41}	0.182

BMI**	32.69 {5.10}	30.88 {6.33}	31.39 {4.62}	0.561

Nationality^^+^^				0.43

Arabic speaking	17 (53.1)	4 (36.4)	7 (63.6)	

Non-Arabic speaking	15 (46.9)	7 (63.6)	4 (36.4)	

Indian (34.6%)	6 (40)	0	3	

Filipino (26.9%)	4 (26.7)	2 (28.6)	1 (25)	

Iranian (11.5%)	3 (20)	0	0	

Sri Lankan (7.7%)	1 (6.7)	1 (14.3)	0	

American (3.8%)	0	1 (14.3)	0	

Others (15.3%)	1 (6.7)	3 (42.8)	0	

Total hospital stay (days)*	13.50 (9.5)	12 (12)	3 (3)	0.000

ICU stay (days)	6 {7.8}	5 {8}	2 {4}	0.030

Vaccination status^+^				0.000

Unvaccinated	32 (100)	11 (100)	1 (9.1)	

Vaccinated	0	0	10 (90.9)	

Chest X-ray^+^				0.020

B/I infiltrates	17 (54.8)	2 (20)	5 (55.6)	

Consolidation	0	1 (10)	1 (11.1)	

Pulmonary edema	0	0	1	

Normal	0	0	1	

Others	14 (45.2)	7 (70)	1 (11.1)	

Anesthesia mode^^+^^				0.330

Spinal	10 (52.5)	5 (62.5)	5 (55.6)	

Epidural	0	1 (12.5)	2 (22.2)	

CSE	1 (5.3)	0	1 (11.1)	

GA	8 (42.1)	2 (25)	1 (11.1)	

Comorbidities^§^				

Asthma	4 (4.2)	0	1 (3)	

Obesity	4 (4.2)	0	1 (3)	

Anemia	11 (11.6)	6 (18.2)	3 (9.1)	

Gestational DM (T1 and tbl2)	14 (14.3)	0	0	

Hypothyrodism	5 (5.3)	1 (3)	0	

Primary HTN	1 (1.1)	0	0	

PET	1 (1.1)	0	0	

Eclampsia	2 (2.1)	0	0	

A-Fib	1 (1.1)	0	0	

Others	2 (2.1)	1 (3)	0	

Symptoms^§^				

SOB	24 (15)	5 (9.1)	0	

Malaise	2 (1.3)	0	4 (7.3)	

Headache	3 (1.9)	2 (3.6)	1 (1.8)	

Loss of smell/taste	1 (0.6)	0	0	

Cough	30 (18.8)	9 (16.4)	2 (3.6)	

Fever	20 (12.5)	7 (12.7)	6 (10.9)	

Diarrhea	4 (2.5)	0	1 (1.8)	

Vomiting	1 (0.6)	2 (3.6)	0	

Body pain	6 (3.8)	0	0	

Others	28 (17.5)	9 (16.4)	1 (1.8)	

Blood group				0.666

A	1 (3.1)	0	0	

A-	3 (9.4)	0	0	

A^+^	5 (15.6)	2 (18.2)	3 (27.3)	

AB^+^	6 (18.8)	1 (9.1)	1 (9.1)	

B^+^	8 (25)	1 (9.1)	3 (27.3)	

O-	1 (3.1)	0	0	

O^+^	8 (25)	7 (63.6)	4 (36.4)	

Blood parameter				

Hb*	11.24 (1.17)	11.12 (1.19)	10.78 (1.63)	

WBC**	5.8 {4}	6.8 {4.10}	10 {18.68}	

CRP**	56.5 {50.1}	48.7 {38}	24 {45.7}	

Creatinine**	39.5 {15.3}	39 {5}	21.12 {37]	

CT value**	21.70 {6.84}	19 {5.50}	22.60 {8.13}	

*Child-related parameters*				

Birth weight**	2.88 {1.37}	2.86 {0.98}	3.14 {1.06}	0.000

APGAR score at 5 min**	10 {1.8}	10 {1.3}	10 {0}	0.542

NICU admission				0.736

No	19 (67.9)	8 (72.7)	8 (72.7)	

Yes	9 (32.1)	3 (27.3)	3 (27.3)	


*N*: number of patients, ^*^mean (standard deviation), ^*^^*^median {interquartile range}, ^+^frequency (% within column), §number (%) based on multiple responses. The bold values are statistically significant at the 95% confidence level (*p* < 0.05).CSE: combined spinal–epidural, GA: general anesthesia, DM: diabetes mellitus, HTN: hypertension, PET: pre-eclampsia, A-Fib: atrial fibrillation.

## Appendix A

[Table tbl6]
